# SARS-CoV-2 and Asbestos Exposure: Can Our Experience With Mesothelioma Patients Help Us Understand the Psychological Consequences of COVID-19 and Develop Interventions?

**DOI:** 10.3389/fpsyg.2020.584320

**Published:** 2020-12-22

**Authors:** Antonella Granieri, Michela Bonafede, Alessandro Marinaccio, Ivano Iavarone, Daniela Marsili, Isabella Giulia Franzoi

**Affiliations:** ^1^Department of Psychology, University of Turin, Turin, Italy; ^2^Occupational and Environmental Medicine, Epidemiology and Hygiene Department, Italian Workers’ Compensation Authority (INAIL), Rome, Italy; ^3^Environmental and Social Epidemiology Unit, Department of Environment and Health, Istituto Superiore di Sanità, Rome, Italy; ^4^WHO Collaborating Centre for Environmental Health in Contaminated Sites, Rome, Italy

**Keywords:** COVID-19, mesothelioma, asbestos, psychological intervention, occupational risk

## Abstract

Since its emergence, the novel coronavirus disease of 2019 (COVID-19) has had enormous physical, social, and psychological impacts worldwide. The aim of this article was to identify elements of our knowledge on asbestos exposure and malignant mesothelioma (MM) that can provide insight into the psychological impact of the COVID-19 pandemic and be used to develop adequate interventions. Although the etiology of Covid-19 and MM differs, their psychological impacts have common characteristics: in both diseases, there is a feeling of being exposed through aerial contagion to an “invisible killer” without boundaries that can strike even the strongest individuals. In both cases, affected persons can experience personality dysfunction, anxiety, depression, and posttraumatic symptoms; helplessness, hopelessness, and projection of destructive thoughts onto external forces often emerge, while defense mechanisms such as denial, splitting, repression, and reduced emotional expression are used by individuals to contain their overwhelming anxieties. We believe that in both diseases, an integrated multidimensional intervention offered by hospitals and other public health services is the most effective approach to alleviating patients’ and caregivers’ psychological distress. In particular, we emphasize that in the context of both MM and COVID-19, Brief Psychoanalytic Group therapy can help patients and caregivers attribute meaning to the significant changes in their lives related to the experience of the disease and identify adaptive strategies and more realistic relational modalities to deal with what has happened to them. We also highlight the importance of developing a surveillance system that includes individual anamnestic evaluation of occupational risk factors for COVID-19 disease.

## Introduction

The novel coronavirus disease of 2019 (COVID-19) emerged at the end of 2019 in China and was classified as a pandemic by the World Health Organization [WHO] (2020a) on March 11, 2020. Since then, it has had an enormous impact on society, forcing health professionals to work long and exhausting shifts, researchers to close their laboratories, teachers to find new ways of delivering education, and workers to adapt to remote working if they have not lost their jobs (Castelnuovo et al., 2020). In many places, local authorities have had to take extreme actions to contain the spread of COVID-19, including lockdown of entire countries. The disease itself, fear of contagion, and measures to contain these have significantly affected people’s occupational and social lives as well as their physical health and psychological well-being. The latter two specifically have been a major challenge for national health systems. In order to develop effective strategies to support patients and their families, it is critical to determine the impact of COVID-19 on their psychological well-being.

As professionals working in close contact with the health system and with specific expertise in contaminated areas across different scientific fields, we have been repeatedly asked whether our experience with exposure to various harmful substances and associated diseases—in particular, asbestos exposure, and malignant mesothelioma (MM)—can be useful for analyzing the psychological impact of COVID-19.

During our decade of work at Contaminated Sites, we have emphasized the need for an integrated approach to the care of patients with MM and their caregivers that involves clinical psychologists and psychotherapists with specific expertise in the field throughout the care process from diagnosis to therapeutic decision-making and up to the end of life (Granieri, 2015, Novello et al., 2016; Granieri et al., 2018). From this perspective, some of the authors have developed a psychological intervention termed Brief Psychoanalytic Group (BPG) therapy that consists of 12 1 h weekly therapeutic sessions for MM patients and their caregivers in the first months following diagnosis (Granieri et al., 2018). To date, this intervention has been implemented only in the National Priority Contaminated Site (NPCS)^1^ of Casale Monferrato, a town in Northwestern Italy that is well-known for the high incidence of and mortality from MM among its residents. The high rates of mortality are associated with exposure to asbestos originating from the Eternit factory, one of the largest asbestos-processing companies in Europe (DeGiovanni et al., 2004; Ferrante et al., 2007; Bertolotti et al., 2008; di Orio and Zazzara, 2013). Our aim in developing BPG therapy was to establish a clinical model that can be adapted to different circumstances and diseases. Therefore, we have been asked whether this type of intervention can be successfully applied to COVID-19 patients as well as their caregivers.

The aim of this article was to identify elements from our findings on communities affected by asbestos exposure, and more specifically MM patients and their caregivers that can offer insight into the psychological impact of the COVID-19 pandemic and guide the development of adequate psychological interventions.

## Epidemiologic Data

The history of epidemiologic surveillance of COVID-19 and MM differs considerably. MM was identified as resulting from asbestos exposure in the 1970s, and its incidence has been monitored by means of a national registry established in the 1990s and implemented since 2002 that includes diagnosed cases since 1993. In contrast, severe acute respiratory syndrome coronavirus 2 (SARS-CoV-2) is a novel virus whose health-related effects in humans were unknown until the emergence of the COVID-19 epidemic in China in late 2019. In Italy, nationwide epidemiologic surveillance of COVID-19 was established in January 2020.

Asbestos-related health effects are a major health concern in Italy, where about 3,748,550 tons of raw asbestos were used in a variety of industrial activities before it was banned in 1992. The Italian mesothelioma registry has documented more than 1,600 incident cases of MM per year and an annual standardized rate of the disease > 3 per 100,000 male inhabitants in recent years (Istituto nazionale per l’Assicurazione contro gli Infortuni sul Lavoro [INAIL], 2018). The median age at diagnosis in occupationally exposed males is 70.5 years, and the male-to-female (M/F) ratio is 2.6 (Marinaccio et al., 2018). The prognosis in MM remains poor. Occupational exposure to asbestos has been ascertained in most cases, and MM is the most prevalent occupational cancer, with an incidence and attributable fraction among cases ranging from 70 to 90% (Rushton et al., 2010).

Italy has one of the highest clinical burdens of COVID-19 in the world; as of November 12, 2020, there were 1,028,424 cases of infection and 42,953 associated deaths in the country (European Centre for Disease Prevention and Control [ECDPC], 2020); 53,276 of these (18.39%) were healthcare workers (Istituto Superiore di Sanità [ISS], 2020a). Of all confirmed cases, the median age at diagnosis is 56 years and the M/F ratio is 1.2.

## MM and COVID-19: Differences and Similarities in Etiology

Table 1 compares MM and COVID-19 symptoms. There are several differences but also some similarities between the two diseases in terms of etiology. One striking similarity is that both MM and COVID-19 are almost always associated with a specific causative agent—i.e., asbestos and SARS-CoV-2, respectively. We previously reported that many inhabitants of asbestos NPCSs besides patients and their families fear aerial contagion by an “invisible killer” (Guglielmucci et al., 2015; Granieri, 2016a). Indeed, occupational and environmental exposure to asbestos involves entire communities. As suggested in previous research, working at a Contaminated Site such as Casale Monferrato implies taking into account each inhabitant’s unconscious representation(s) for choosing to continue residing in a place that is now recognized as potentially dangerous, their awareness of being exposed to an environment contaminated as a result of a profit-driven logic, and the corruptive and collusive dynamics of the relationship with a factory that has for years contributed to the economic prosperity of the community (Borgogno et al., 2015; Granieri, 2016a, 2017). Inhabitants of NPCSs can harbor intense feelings of guilt or shame, which increases the burden of an already traumatic situation. This is similar to the experience of the COVID-19 pandemic (Benziman, 2020; Nana et al., 2020) in which communities are fearful of contagion and worried about spreading the virus to their families, friends, or colleagues. The potential for each individual to be a vehicle of virus transmission can elicit blame, remorse, and regret. At the same time, people may engage in repulsive or isolationist behaviors toward those who are infected. Indeed, the way the community responds to a disease affects how people with the illness feel and behave: when an illness is viewed as something shameful, the affected person may experience more intense feelings of guilt and is at greater risk of social withdrawal.

**TABLE 1:** Comparison between COVID-19 and MM symptoms.

Symptoms	COVID-19	MM
Fever	V	V
Dry cough	V	V
Loss of smell and taste	V	V
Headaches	V	V
Aches, muscle pains	V	
Sore throat	V	V
Fatigue	V	V
Chills, repeated shaking	V	
Diarrhea, vomiting	V	V
Runny nose	V	
Sneezing	V	
Weight loss		V
Dyspnea	V	V
Chest pain	V	V
Swallowing difficulties	V	V
Back Pain		V

**American Society of Clinical Oncology (2017) and World Health Organization [WHO] (2020b).**

Both MM and COVID-19 affect the respiratory system. The most common organ affected by MM is the pulmonary pleura, and its signs and symptoms include shortness of breath due to fluid around the lungs, abdominal swelling, chest wall pain, cough, and fatigue (Moore et al., 2010; American Cancer Society [ACS], 2016). Similarly, > 90% of COVID-19 cases hospitalized in Italy showed pneumonia and respiratory failure, with the most common symptoms being fever, dyspnea, cough, and fatigue (Brioni et al., 2020; Lomoro et al., 2020). However, the two diseases differ in their etiology. Asbestos fibers are released into the atmosphere and inhaled; although these may have a natural origin, anthropogenic activities are the predominant source of atmospheric asbestos fibers (International Agency for Research on Cancer [IARC], 2012). In contrast, SARS-CoV-2 is a virus that is transmitted through direct contact with respiratory droplets of an infected person that are expelled through speaking, coughing, and sneezing. Less frequently, infection can occur through contact with a surface contaminated with the virus (Li Q. et al., 2020; Rothe et al., 2020).

This highlights one of the most significant differences between MM and COVID-19. The former is non-contagious and is associated with environmental or, more frequently, occupational exposure to asbestos and primarily affects workers in asbestos-cement plants and other industrial settings (Vimercati et al., 2019; Catelan et al., 2020). However, MM can also affect anyone living in an asbestos NPCS or who comes into contact with asbestos fibers carried by another person (e.g., in a domestic context such shared living quarters). Indeed, MM has been diagnosed in wives/relatives of workers who were occupationally exposed to asbestos through inhalation of fibers attached to surfaces and contaminated clothes and handling of asbestos waste (Ferrante et al., 2007; Noonan, 2017). This can lead to a feeling of danger in relationships, particularly within a family (Granieri et al., 2018; Padilha Baran et al., 2019). Living in an environment where the risk of exposure to the toxic agent is omnipresent, together with the awareness of the large number of victims and outcome of MM, can cause individuals to enter a state of social and emotional detachment (Guglielmucci et al., 2014; Kozlowski et al., 2014), which is similar to the feelings aroused by COVID-19.

COVID-19 is a transmissible disease that is mostly linked to exposure in living environments, especially indoor and crowded places (Parvin et al., 2020; Zhao et al., 2020). In some cases, adult sons or daughters are responsible for infecting their parents, who are more likely to have fragile health (Mapelli, 2020). From a clinical standpoint, this can lead to the feeling of being a harbinger of death, especially in those who have infected elderly parents; this is similar to what we have encountered in many MM patients living in asbestos NPCSs, who report being stigmatized as a “plague spreader” by the community and experiencing feelings of intense guilt for having contaminated their families by transporting a toxic agent from the workplace into their home (Guglielmucci et al., 2018; Wang et al., 2020).

COVID-19 is an ongoing challenge for occupational health (Burdorf et al., 2020), with several work environments associated with high risk of viral transmission. During the epidemic, the Italian National Workers Compensation Authority (INAIL) introduced the notion of work-related SARS-CoV-2 infection as an occupational injury and processed compensation claims from workers all over the country including healthcare and public administration workers, nursing home staff, and workers in other economic sectors. In the case of COVID-19 but not MM, the occupational source of the disease is primarily related to the provision of care to affected patients (Roggli et al., 2002; Koh, 2020; Lewandowski, 2020; Ng et al., 2020). Exposure to SARS-CoV-2 varies according to the work environment and the employee’s occupational role (Bai et al., 2004; Brooks et al., 2018). Healthcare workers have very high exposure to the virus as they are in constant contact with infected individuals, making theirs a high-risk occupation in terms of the impact on their mental health, especially during a pandemic (Bai et al., 2004; Maunder, 2004; Chen et al., 2005; Maunder et al., 2006; Wu et al., 2009; Hamouche, 2020; Ho et al., 2020; Huang and Zhao, 2020; Huang et al., 2020; Koh, 2020; Xiang et al., 2020; Zhu et al., 2020).

Despite their distinct etiologies, MM and COVID-19 may have a similar psychological impact on individuals: inhabitants of asbestos NPCSs often report a feeling of being exposed without protection to a threat in their living environment while in a broader sense, a virus such as SARS-CoV-2 does not have any geographic boundaries (Granieri, 2016a; Consumer News and Business Channel [CNBC], 2020) as anyone can be infected, and there can be a new infection or death at any moment of any day (Lazzerini and Putoto, 2020; Montemurro, 2020). The idea of a “phantom of death” that can strike even the strongest person has been expressed by inhabitants of asbestos NPCSs (Granieri, 2016a). This is still relevant to COVID-19, although it is less deadly than MM and is most lethal in vulnerable populations. Thus, some people who have not contracted COVID-19 consider their home as their only refuge, and it remains so only through avoidance of all contact with the outside world. This can lead to a self-confinement that persists even after the lockdown imposed by authorities is lifted (Banerjee and Rai, 2020).

Another important difference in the etiologies of MM and COVID-19 is that asbestos is linked to industrial production and human activities, whereas SARS-CoV-2 is an infectious agent with a natural origin. Humans are facing an increasing number of contaminants associated with manufacturing (Goldsteen and Schorr, 1982; Cline et al., 2008, 2014). Epidemics related to pathogens such as viruses or bacteria are an archaic threat but are unexpected and frightening, especially in Western countries. While asbestos exposure can be blamed on specific people or interests, the same is not true for COVID-19. A calamity without anyone to hold responsible is difficult to accept, and when it cannot be attributed to a known source, people tend to protect themselves from their fears and sense of helplessness by ascribing the problem to a more familiar and controllable cause out of a psychological need to identify a culprit. This can include scientific laboratories, China, 5G, governments, the healthcare system, God, or even other people who do not behave as they think appropriate for containing the spread of the virus. In this context, myths and misconceptions (“fake news”) about COVID-19 have emerged especially on the internet, which has fueled anxiety among people (Barreneche, 2020), forcing national and international public health agencies to counter the propagation of misinformation by refuting false claims and communicating correct information (Italian Ministry of Health [IMH], 2020; Istituto Superiore di Sanità [ISS], 2020b; World Health Organization [WHO], 2020a,b).

There are important similarities and differences between MM and COVID-19 in terms of the affected populations and disease onset time. The interval from exposure to asbestos to the development of symptoms in MM is on average ≥ 40 years; as such, this cancer mainly occurs in adults and older people (Marinaccio et al., 2007; Barone-Adesi et al., 2012; Reid et al., 2014). In contrast, people of any age can be infected with COVID-19, although older people are more vulnerable (Niu et al., 2020). Thus, older people have a higher risk of both diseases, but with a fundamental difference in timing: MM has a long latency period, whereas the latency of COVID-19 ranges from a few days to 2 weeks (Lauer et al., 2020). A diagnosis of either disease can be traumatic, but the trauma of COVID-19 is current while that of MM is rooted in the past—i.e., people are paying the price for decisions that were made and events that unfolded many years prior.

Workers can limit their exposure to asbestos by using personal protective equipment (e.g., masks, gloves, and suits); additionally, the risk of exposure for workers and the general public can be minimized by providing information and promoting awareness of the health-related effects and through compliance with Italian law 257/1992 and related legislation that prohibits production involving asbestos and requires the implementation of asbestos remediation processes. The spread of COVID-19 can likewise be limited by providing information on the risks and health-related effects, thereby increasing public awareness of preventative measures and promoting the adoption of health-protective individual behaviors (e.g., hand-washing, wearing personal protective equipment, and social distancing and restrictions to outdoor gatherings) (Hellewell et al., 2020; Liu et al., 2020; Xu et al., 2020). However, these measures undermine a fundamental need of humans to socialize and engage in relationships, as well as norms of reciprocity, social trust, and support; they also require individuals and communities to utilize adaptive resources that rely on bonding, bridging, and linking aspects of social capital to respond to public health threats (Szreter and Woolcock, 2004) and pandemics (Chuang et al., 2015; Pitas and Ehmer, 2020). Although digital technology may enable the preservation of social connections during physical distancing, complete physical isolation (which can be quarantine or shielding in some contexts) can affect people’s emotional well-being and sense of self and life purpose (Brioni et al., 2020).

Various drugs have been tested for the treatment of MM, but to date there is no cure and the prognosis is poor. Vaccines for COVID-19 are under development but are not yet available. However, it should be noted that the mortality rate of COVID-19 is around 10%; that is, patients recover in most cases.

## Psychological Impact of Asbestos Exposure and SARS-CoV-2 Infection

### Factors Negatively Affecting Mental Health

Asbestos exposure as well as SARS-CoV-2 infection has lasting and negative consequences for both physical and mental health (Taylor et al., 2008; Guglielmucci et al., 2015; Adhanom Ghebreyesus, 2020). Psychological effects of MM and COVID-19 are observed not just in patients and caregivers, but in the entire exposed population (Table 2).

**TABLE 2:** Psychological impact of COVID-19 and MM on exposed population, affected patients, and their caregivers.

Psychological impact	COVID-19			MM		
		
	Exposed population	Patients affected	Caregivers	Exposed population	Patients affected	Caregivers
Depression	44.1*^%k^*	29.2%*^p^*	62.5%*^e^*,*^r^*	V^b,c^	19%*^o^*	V^b,c,^*^h^*
Anxiety	32.4%*^k^*	20.8%*^p^*	20.5%*^e^*	V^b,c^	32%*^o^*	V^b,c,^*^h^*
Stress	37%*^k^*		36.4%*^e^*	V^b^	39.8%*^o^*	V^b,c,^*^h^*
Fear	V*^i^*	V*^i^*		V^b,c^	V*^g^*	
Worries	V*^i^*	V*^i^*				
Anguish	V*^i^*	V*^i^*	V*^e^*	V^b,c^	V*^g^*	V^b,c^
Fatigue	V*^n^*		V*^e^*	V^b,c^		
Loneliness	35.86%*^i,l,p^*	35.86%^i^				
Social withdrawal	V*^a,i^*			V^b^	V*^g^*^,c,b^	V^b,^*^h^*
Difficulty of adaptation	V*^f,j^*	V*^j^*			V*^g^*^,c^	
Hopelessness					V*^g^*^,b^	V*^h,c^*
Intrusive thoughts					V^b,c^	
Uncertainty	V*^f^*	V*^j^*		V^b,c^	V^b,^*^c^*	V^b,c^
Loss of control	V*^l^*	V*^j^*	V*^e^*	V^b^	V*^g^*	V^b^
Compromised sense of belonging	V*^j^*	V*^j^*				
Somatization	7,4%*^m,q^*	7,4%*^m,q^*		V^b^	V*^c^*	V^b,^*^h^*

*^*a*^Banerjee and Rai (2020), ^*b*^Bonafede et al. (2018), ^*c*^Bonafede et al. (2020), ^*d*^Burhamah et al. (2020), ^*e*^Dhiman et al. (2020), ^*f*^Furlong and Finnie (2020), ^*g*^Hughes and Arber (2008), ^*h*^Kleine et al. (2019), ^*i*^Li and Wang (2020), ^*j*^Monterrosa-Castro et al. (2020), ^*k*^Odriozola-González et al. (2020), ^*l*^Rubin and Wessely (2020), ^*m*^Shangguan et al. (2020), ^*n*^Townsend et al. (2020), ^*o*^Ugalde et al. (2012), ^*p*^Xiang et al. (2020), ^*q*^Zhang J. et al. (2020), ^*r*^Zhong et al. (2020). *V* = *Aspects cited, but without prevalence percentage.**

### Asbestos Exposure

For the general population living in NPCSs, the traumatic experience is linked to the workplace but affects the entire community because of the hazards associated with their living place. People who have experienced this type of collective trauma exhibit personality dysfunction, anxiety, and depression, as well as an increased frequency of dissociative experiences, somatization, and enactment entailing many somatopsychic features (Borgogno et al., 2015; Granieri, 2016a; Colizzi et al., 2020). People living in NPCSs have had to find a balance between immunitas (isolation and enclosure within one’s own identity boundaries to protect oneself from contamination by others) and communitas (opening up one’s life to others and facing the fear of contagion in the encounter with the other’s specificity) (Granieri, 2013, 2016b; Guglielmucci et al., 2015). One study of MM patients found that 32% had clinical or subclinical anxiety, 19% had depression, and 39.8% reported stress (Ugalde et al., 2012). In particular, psychological distress was linked to “Dealing with concerns about your family’s fears and worries” (62%). Social isolation resulting from depression, apathy, and stigma is an issue faced by patients and caregivers (Hughes and Arber, 2008; Guglielmucci et al., 2018). MM patients reported a high level of frustration and emotional distress in reaction to physical symptoms (Hughes and Arber, 2008). MM has a unique psychosocial impact because of the high symptom burden, incurability, rarity, and asbestos-related etiology (Bonafede et al., 2018). Awareness of the work-related origin of the disease leads to specific forms of emotional distress in MM patients such as anger, anguish, and worry, along with a sense of guilt for having risked their families’ health (Braun and Kisting, 2006; Guglielmucci et al., 2014). These negative emotions are difficult to express, and MM patients—as well as other people who are exposed to asbestos—may experience loss of their sense of belonging and control (Guglielmucci et al., 2018).

### SARS-CoV-2 Infection

A pandemic can place a severe strain on society’s mental health resources, potentially leading to untreated mental health issues (Douglas et al., 2009; Banerjee, 2020; Yang et al., 2020). Fear of infection is a common reaction to pandemics; the possibility of infection can cause pervasive worry over one’s health and potential to infect others, especially family members (Bai et al., 2004; Desclaux et al., 2017). People in high-transmission areas may exhibit personality dysfunction, anxiety, and depression, and somatic disorders may be exacerbated by the intense fear of infection (Mohammed et al., 2015; Ornell et al., 2020; Vigo et al., 2020; Xing et al., 2020). Symptoms of posttraumatic stress disorder or complicated grief disorder are also sequelae of global emergencies or disasters (Eisma et al., 2019, 2020; Colizzi et al., 2020; Fekih-Romdhane et al., 2020; Tang et al., 2020). Mental health issues during a pandemic are related to the adverse effects of prolonged social distancing, social isolation, and quarantine, and the illness and loss of loved ones (Garety et al., 2001; Wang et al., 2017; Bao et al., 2020; World Health Organization [WHO], 2020c). Stress, anxiety, depression, and insecurity have emerged over the many months of the COVID-19 pandemic, even in non-infected individuals (Duan and Zhu, 2020); the overall prevalence of depressive symptoms, anxiety, and stress symptoms among adults was found to be 44.1, 32.4, and 37%, respectively (Odriozola-González et al., 2020). Anxiety can be accompanied by feelings of anguish, social isolation, panic, and irritation; inability to concentrate; and sleep disturbance (Monterrosa-Castro et al., 2020). One study reported that 7.4% of the population showed various somatic symptoms related to chronic stress caused by the COVID-19 pandemic (Zhang J. et al., 2020). Unemployment, uncertainty, distress, the increasing death toll, and restrictions imposed by lockdown can cause a strain on mental health (Banerjee and Rai, 2020). Higher rates of psychological symptoms such as emotional disturbance, depression, and posttraumatic stress were reported in people under quarantine than in those who had not been quarantined (Brooks et al., 2020). Moreover, in the case of mass quarantine, social isolation, worry, and an inability to tolerate distress can exacerbate anxiety and feelings of being trapped and losing control (Rubin and Wessely, 2020).

Although a pandemic affects the entire population, individuals who have experienced potentially traumatic events such as a threat to one’s own health or loss of a loved one or their livelihood have a higher risk of developing mental health problems (Inchausti et al., 2020; Li Z. et al., 2020; Lima et al., 2020; Sritharan and Sritharan, 2020). Even families who have not been in direct contact with SARS-CoV-2 may experience indirect effects of the pandemic (Van Bavel et al., 2020). The drastic change in daily habits and restrictions on movement and activities in order to limit virus transmission may enhance worry and insecurity. Families are more likely to experience increasing social isolation, which may further exacerbate distress and increase susceptibility to stress, which can have harmful effects on both mental and physical health (Hawkley and Cacioppo, 2010). With the pervasive news coverage of the exponential spread of COVID-19, some people may wonder whether they or a loved one will inevitably contract or even die from the virus (Bertuccio and Runion, 2020; Wallace et al., 2020). An increased prevalence of depression (29.2%) and anxiety (20.8%) was observed in patients who had experienced COVID-19 infection (Zhang J. et al., 2020), and the incidence of fatigue 10 weeks after initial COVID-19 symptoms was 52.3% (Townsend et al., 2020). Patients may be fearful of the consequences of the infection, and those in quarantine may experience boredom, loneliness, and anger (Xiang et al., 2020). The rate of loneliness in a sample of people exposed to and affected by COVID-19 was reported to be 35.86%, and people who had COVID-19-related symptoms were more likely to develop psychiatric disorders and experience loneliness (Li and Wang, 2020).

For most individuals, the COVID-19 crisis has become a new normal. People are often confined in their homes in some cases unable to work and may feel cut off from close friends and family. The extreme social restrictions as well as the emergency situations that healthcare professionals face daily require that individuals make psychosocial adjustments to the long-lasting and substantial impact of the disease on their lives. Distress and loneliness can profoundly affect people’s perception of events (Murthy, 2017; Berg-Weger and Morley, 2020; Cerami et al., 2020; Zandifar and Badrfam, 2020). A recent study showed that health professionals working to fight COVID-19 were more severely affected by mental health issues (Dai et al., 2020; Lai et al., 2020; Zhang W. et al., 2020; da Silva and Neto, 2021) and indirect traumatization (Li Z. et al., 2020) than other occupational groups.

### Caregiver Burden

A diagnosis of MM often requires the intervention of a family member, who assumes the role of caregiver and represents the support structure for the patient through the various stages of the disease (Hughes and Arber, 2008). This requires a radical reassessment of the primary needs within the family and can involve drastic changes in daily activities, work, and relationships and reformulation of roles within the family. Such conditions can lead to significant distress (Maguire, 1985; Frank, 1991). Caregivers of MM patients are prone to experiencing depression, anxiety, hopelessness, somatic symptoms, social isolation, and financial stress (Adelman et al., 2014; Kleine et al., 2019). It was recently shown that caregivers of MM patients were more severely traumatized than the patients themselves, reporting higher frequencies of intrusive thoughts about death and exhibiting physiologic hyperactivation (Bonafede et al., 2020). The risk of depression is associated with use of avoidance strategies in caregivers, with a higher risk in females as compared to males (Bonafede et al., 2020).

The COVID-19 pandemic is already exacerbating caregiver responsibilities especially among women, with schools and childcare centers preventatively closed nationwide (Graves, 2020). Caregivers of COVID-19 patients face many challenges (Gulia et al., 2020). A recent study showed that long-term caregivers were more likely than short-term caregivers to have had a mental health condition prior to the COVID-19 pandemic, and both groups were more likely than non-caregivers (Park, 2020). Long-term caregivers also developed more somatic symptoms, and the rates of depression, anxiety, and stress symptoms in this group were 62.5, 20.5, and 36.4%, respectively (Dhiman et al., 2020). Psychological distress in caregivers is also exacerbated by social distancing and restrictions to individual mobility and social activities, as low social support is associated with higher levels of worry and depression (Zhong et al., 2020).

### Effects of Isolation

Isolation and social distancing have affected MM and COVID-19 patients in different ways. As noted above, MM patients often report feeling stigmatized as “plague spreaders” by the community (Granieri, 2013; Guglielmucci et al., 2014), and their caregivers also experience social isolation. The COVID-19 pandemic has added a new dimension to this isolation both at home and in hospitals. In order to prevent transmission, COVID-19 patients are usually admitted to a separate ward from other hospitalized patients and face a prolonged period of quarantine after discharge. Patients often die alone, and family members are alone in their mourning, without the possibility of experiencing the moments of death and the start of the mourning process. In some instances, people have been cremated because of a lack of space even if this was not their wish before their death, such that relatives were deprived of the last chance to see their loved one’s body in repose. In other cases, people have been buried wearing only a white sheet, reminiscent of past ages when life was given a lesser value. The negative psychological repercussions of isolation are further complicated by the fact that people in need do not always seek help if they perceive that experiencing pain after a collective traumatic experience or after isolation is common (Meda and Slongo, 2020; Samson, 2020).

### Defense Mechanisms

Common responses to traumatization—which depend on the mind’s degree of integration and quality of functioning—range from a frozen affective internal state based on dissociation and denial, to resilient behaviors developed following exposure to the traumatizing event (Granieri et al., 2018; Bonafede et al., 2020). Defense mechanisms in inhabitants of asbestos NPCSs are mainly aimed at maintaining, protecting, modifying, or repairing the shared group identity. Denial, splitting, repression, and reduced emotional expression allow individuals to contain their overwhelming anxiety and profound feelings of shame and guilt associated with the fact that they have accepted something dangerous to themselves and their families (Granieri, 2016b). Indeed, although asbestos production had many benefits, in time it became evident that it endangered the population and would continue to harm those residing in the NPCS (Guglielmucci et al., 2014). Inhabitants of asbestos NPCSs often direct their rage toward asbestos, institutions, delays in diagnosis, experimental protocols, or the complicated process of applying for financial compensation (Guglielmucci et al., 2015, 2018). This projection of destructive feelings onto external objects is a self-protective measure against internally directed anger (e.g., frustration at being incapacitated as a result of the disease, fear of impending mourning and death, or the feeling of not being understood) or attempts to conceal feelings of deep depression (e.g., discouragement, sadness, and helplessness) (Granieri and Borgogno, 2014; Granieri, 2015; Granieri et al., 2018). It is likely that that people exposed to asbestos and SARS-CoV-2 experience similar feelings of helplessness and hopelessness and have aggressive cogitations directed toward the source of the trauma. As in the case of inhabitants of asbestos NPCSs, during a pandemic people rely on defense mechanisms such as denial, splitting, repression, reduced emotional expression, and a frozen affective state. For example, at the time of writing this article, some people were still denying the risk of COVID-19 and refused to adhere to government-ordered protective measures. On the contrary, others are so fearful that they have adopted obsessive-compulsive defenses (i.e., cancelation) to avoid infection. The rapid evolution of the pandemic (Cucinotta and Vanelli, 2020), lockdown measures (Wilder-Smith and Freedman, 2020), and reports of fatal outcomes (Onder et al., 2020) may unintentionally encourage societal over-concern, which can degenerate into heightened anxiety and stress responses and the development of fictitious symptomatology, leading to misguided health-protective and help-seeking behaviors (Garfin et al., 2020).

### Experience of MM Patients During the COVID-19 Pandemic

The British Thoracic Society recently published recommendations on COVID-19 and lung cancer/mesothelioma (British Thoracic Society [BTS], 2020). As the latter patients are at risk of a fatal outcome if they contract COVID-19, particularly strict restrictions to their movements and social interactions are necessary. This can magnify the sense of isolation that they experience as MM patients, resulting in not just an additive but a synergistic interaction between the impact of their existing disease and that of the measures they have to follow to prevent another potentially fatal disease (Golden, 2020; Mannino, 2020).

## Psychological Interventions in Response to COVID-19 Based on Experiences With MM Patients and Their Caregivers

Specific preventive strategies at the community level must be provided to mitigate the psychological and psychosocial impact of the COVID-19 pandemic. Our experience with MM patients and their caregivers has highlighted the need for an integrated intervention that restores in patients a sense of control over and responsibility for their health and treatment and reduces psychological distress in both patients and caregivers, while providing them with strategies to actively face the disease (Granieri, 2015). This is also important for addressing the COVID-19 pandemic (Orrù et al., 2020; Salari et al., 2020).

Psychoanalysis has been increasingly focused on large-scale disasters (e.g., natural catastrophes, pandemics, accidents, war, and so on) and their devastating physical, psychological, and relational impacts (Rosenbaum and Varvin, 2007; Boulanger et al., 2013; Granieri, 2016a), with therapeutic techniques adapted to specific contexts without overshadowing the basic theoretical model (Ferenczi, 1928). The BPG intervention designed by some of the authors of this paper for MM patients and their caregivers in the asbestos NPCS of Casale Monferrato (Granieri et al., 2018) can also be applied to COVID-19 patients.

We believe that BPGs can be particularly useful in those situations where very intense feelings compromise the possibility to give meaning to the experience of the disease. In both the context of MM and COVID-19, we propose that BPG interventions can help patients and their caregivers give meaning to the significant changes in their lives connected to the experience of the disease and the therapies, allowing them to identify more adaptive strategies and more realistic relational modalities to deal with what has happened to them.

BPG therapy consists of 12 1-h weekly group sessions led by two psychoanalytically oriented psychotherapists with specific expertise in this field. The time-limited intervention was designed for the first months following diagnosis, given that this period is characterized by intense traumatization, disintegration, splitting, and posttraumatic stress (Arber and Spencer, 2013; Guglielmucci et al., 2018), and considering the limited life expectancy and rapid deterioration of health of MM patients after the diagnosis. Similarly, the traumatic experiences of COVID-19 patients and caregivers related to the disease, quarantine, and care process must be addressed as soon as possible, even though video conferencing, given the physical condition of affected patients.

In both MM and COVID-19, the traumatic experiences of patients have an impact on caregivers. Thus, an intervention aimed at both groups may reveal unconscious processes connected to the overwhelming impact of the disease and the death anxieties aroused. Patients and caregivers are encouraged to project the traumatic emotions they are experiencing and can be helped in elaborating what is happening to them, in facing the fear of loss and death, and can receive help in translating their dissociated traumatic experience into thoughts and words (Granieri, 2016a, 2017; Granieri et al., 2018). Moreover, the presence of other families with different psychological and relational characteristics allows participants to experience within the group that there are different strategies and processes—which may be more or less mature or dysfunctional—for managing the disease, as well as their physical and psychological consequences (García Badaracco, 2000; Granieri and Borgogno, 2014). Finally, co-conduction of the sessions by two psychotherapists with a specific clinical and professional expertise ensures greater focus on unconscious emotional content and simultaneous consideration of the intrapsychic, intrafamily, and interpersonal dynamics of the group.

BPG therapy progresses through different phases, each with specific aims (Granieri et al., 2018). In the initial phase (sessions 1–3), clinicians explore through narratives shared with the group how people relate to an ill body and its new needs, as well as anxieties and unconscious affect related to the danger of living in a contaminated site. In this phase, the co-conductors help the group identify a shared and recurring topic [somatopsychic focus (SPF)], a shared image/metaphor that links physical symptoms with emotions, feelings, and fantasies. In the central phase (sessions 4–8), the co-conductors help the group address the SPF through recognition of feelings and fantasies and their connection to their daily life experiences such as physical symptoms and medical treatments. Finally, the concluding phase (sessions 9–12) helps the group reconsider the narrative of the disease including its development among members, eventual absences or deaths, emotions shared with the group, and strategies used to face the disease. Additionally, the group explores fantasies about the end of the therapy and identifies what each member will take away from the work that was done during the sessions.

Under traumatic circumstances, the flow of time can sometimes collapse, leaving the individual stuck in the traumatic event and prematurely withdrawing from a life that has lost its appeal and attractiveness, becoming a sort of black-and-white photograph (Freud, 1915). BPG therapy can be particularly useful in situations where very intense feelings undermine the possibility of ascribing meaning to the experience of the disease. In the contexts of MM and COVID-19, BPG therapy can help patients and caregivers find meaning in the significant changes in their lives related to the disease and medical treatments, allowing them to identify more adaptive strategies and more realistic relational modalities to deal with their situation. Thinking together within the group may help participants to give meaning to the transformations in their lifestyle brought about by the experience of the disease and the related feelings. The psychotherapy group therefore represents a setting where listening of the specific personal modalities through which despair and helplessness related to the catastrophic impact of the diagnosis are expressed, allows to share, allowing the participant to regain their self-confidence as well as trust in others and hope in life (Ambrosiano, 2016).

## Conclusion

MM and COVID-19 have different etiologies but are similar in some important respects, including a clear relationship with their etiologic agents, symptomology, and target organ (respiratory system), and psychological impact. We propose applying what we have learned from MM—specifically, the impact on patients, caregivers, and the general population—to the new challenge of COVID-19. The occupational origin of the disease (predominant in MM but still relevant in COVID-19) suggests the need to develop a surveillance system that includes an individual anamnestic evaluation of occupational risk factors for COVID-19. An occupational surveillance system was recently proposed for monitoring and preventing SARS-CoV-2 transmission in workplaces and improving the effectiveness of the insurance system (Marinaccio et al., 2020a). It will also be important to implement a control system that gives adequate consideration to the occupational dimension of risk to correctly manage vaccination policies (Marinaccio et al., 2020b). The BPG therapy model of intervention developed for MM patients and their caregivers can be successfully adapted to COVID-19 patients and caregivers. Living in asbestos-contaminated sites and in areas most affected by the pandemic both have important impacts on different levels of personal experience including being (health and somatopsychic well-being), belonging (the sense of being part of a community), and becoming (one’s expectations for the future) (Fauci et al., 2012; Granieri, 2015). Thus, implementation of an integrated multidimensional intervention by hospitals and other public health services will be useful for addressing the psychological distress and needs of patients and caregivers affected by COVID-19.

## Author Contributions

AG conceived of the presented idea. AG, IF, and MB contributed to the interpretation of the data, drafting and critical revision of the manuscript, giving an important clinical, and intellectual contribution. AM, II, and DM contributed to the interpretation of the data and critical revision of the manuscript. All authors agree to be accountable for all aspects of the work in ensuring that questions related to the accuracy or integrity of any part of the work are appropriately investigated and resolved. All authors contributed to the article and approved the submitted version.

## Conflict of Interest

The authors declare that the research was conducted in the absence of any commercial or financial relationships that could be construed as a potential conflict of interest.

## Footnotes

1 National Priority Contaminated Sites are areas identified based on analysis of contaminating agents as those that are a health and ecologic hazard, have an environmental impact, and cause damage to cultural and environmental assets (Legislative Decree 22/97; Ministerial Decree 471/99; Legislative Decree 152/2006).
